# Stress-Induced Epstein-Barr Virus Reactivation

**DOI:** 10.3390/biom11091380

**Published:** 2021-09-18

**Authors:** Daniel G. Sausen, Maimoona S. Bhutta, Elisa S. Gallo, Harel Dahari, Ronen Borenstein

**Affiliations:** 1 Department of Microbiology and Molecular Cell Biology, Eastern Virginia Medical School, Norfolk, VA 23501, USA; SausenDG@EVMS.EDU (D.G.S.); BhuttaM@EVMS.EDU (M.S.B.); 2 Pinnacle Dermatology, Barrington, IL 60010, USA; esgallomd@hotmail.com; 3 The Program for Experimental and Theoretical Modeling, Division of Hepatology, Department of Medicine, Stritch School of Medicine, Loyola University Chicago, Maywood, IL 60153, USA; hdahari@luc.edu

**Keywords:** EBV, latency, reactivation, stress, vaccination

## Abstract

Epstein-Barr virus (EBV) is typically found in a latent, asymptomatic state in immunocompetent individuals. Perturbations of the host immune system can stimulate viral reactivation. Furthermore, there are a myriad of EBV-associated illnesses including various cancers, post-transplant lymphoproliferative disease, and autoimmune conditions. A thorough understanding of this virus, and the interplay between stress and the immune system, is essential to establish effective treatment. This review will provide a summary of the interaction between both psychological and cellular stressors resulting in EBV reactivation. It will examine mechanisms by which EBV establishes and maintains latency and will conclude with a brief overview of treatments targeting EBV.

## 1. Introduction

Epstein-Barr virus (EBV), also known as human herpesvirus 4, is a widely prevalent pathogen that infects 90% or more of the world population [1,2,3]. Infection most commonly occurs following exposure to contaminated oral secretions, although symptoms do not appear for approximately six weeks, if at all [1]. EBV establishes lifelong latency in infected lymphocytes following acute infection. It can reactivate under appropriate conditions, namely those associated with diminished cell-mediated immunity [4].

The primary disease associated with EBV is infectious mononucleosis. This illness is most commonly seen in adolescents and young adults and presents with fatigue, fever, pharyngitis, cervical lymphadenopathy, and lymphocytosis [5,6]. EBV has been associated with other diseases including chronic fatigue syndrome, Epstein-Barr virus-related post-transplant lymphoproliferative disease, multiple sclerosis, encephalitis, cerebellar ataxia, Alzheimer’s disease, oral hairy leukoplakia, and autoimmune conditions such as Grave’s Disease, Sjögren’s syndrome, and rheumatoid arthritis [6,7,8,9,10,11,12,13,14]. Notably, there is a well-established connection between EBV and malignancies including Hodgkin’s lymphoma, diffuse large B-cell lymphoma, Burkitt lymphoma, primary central nervous system lymphoma, T cell lymphoma, certain gastric carcinomas, and nasopharyngeal carcinoma (NPC) [15,16,17,18]. In fact, it is estimated that EBV causes 1.8% of all cancer-related deaths worldwide [19], while a more recent study found that 265,000 cases of Burkitt lymphoma, Hodgkin’s lymphoma, NPC, and gastric carcinoma alone were caused by EBV in 2017 [20]. There is no vaccine for EBV and current anti-EBV agents are suboptimal due to low potency or high toxicity [21,22].

EBV is a member of the gammaherpesvirus subfamily of herpesviridae. Its virion structure is similar to other herpesviridae and includes a double-stranded DNA core, a surrounding icosahedral capsid, a tegument, and an envelope studded with glycoproteins [23,24]. The tegument encompasses the area between the capsid and envelope [23]. The proteins found therein are involved in numerous viral processes, including reactivation [25], viral envelopment [26], and immune evasion [27,28]. Glycoproteins play an integral role in viral fusion and have also been implicated in immune system evasion [29].

EBV’s ability to infect B cells and epithelial cells is well established [30,31]. The core fusion machinery required for viral entry include the EBV glycoproteins (g) gB, gH, and gL [32]. In B cells, gH and gL complex with gp42 to form a gH/gL/gp42 heterotrimer that is necessary for entry [33]. gp42 is able to interact with human leukocyte antigen (HLA) class II molecules on B cells to trigger viral fusion [32]. gp220/350 tethers EBV to B cells via interactions with complement receptor type 2 (CD21) [34]. In epithelial cells, gH and gL form a heterodimer that can bind the epithelial cell integrins αVβ5, αVβ6, or αVβ8 in the early stages of viral entry [33]. BMRF2, another EBV glycoprotein, likewise interacts with cellular integrins, specifically α1, α5, α3, and αv integrins, to facilitate infection of polarized epithelial cells [35]. Other cellular factors that have been identified as important in EBV epithelial cell entry include neuropilin 1, which interacts with gB [36], ephrin receptor A2, which interacts with gH/gL and gB [37], and non-muscle myosin heavy chain IIA, which interacts with gH/gL [38].

The role of gp220/350 in epithelial cell infection is not as well established, and conflicting reports exist regarding its utility [39,40]. Notably, deleting gp220/350 did not completely abrogate EBV entry into numerous examined cell lines, including human B cells, lymphoid lines, and the majority of epithelial cell lines, although infection was not as efficient in the absence of gp220/350. This indicates that gp220/350 is not necessarily required for infection of either epithelial or lymphocyte cell lines [41]. EBV’s ability to infect T cells is less studied, though it was recently shown that CD21 is important in T cell entry [42]. EBV is also capable of infecting NK cells either by direct viral episome transfer [43] or by a CD21-dependent mechanism. In the latter case, NK cells targeting infected B cells temporarily gain CD21 molecules through synaptic transfer. This allows EBV to bind to and infect the NK cell [44]. A more thorough review of EBV tropism can be found elsewhere [45,46].

In vitro, EBV is capable of infecting numerous cell lines. Examples of B cell lines shown to sustain EBV infection include lymphoblastoid, P3HR1, Rael, Akata, Raji, Daudi, and B95-8 cells, while examples of epithelioid cell lines sustaining EBV infection include GT38, PN, the nasopharyngeal carcinoma line C666, and the gastric carcinoma line AGS [47,48,49,50,51]. EBV has also been shown to infect monocytes [52]. Indeed, an EBV-infected monocyte cell line called E1 has been established [53]. EBV’s ability to infect neuronal cells was established by Jha et al., who were able to successfully infect the neuroblastoma cell line Sh-Sy5y, neurons from the teratocarcinoma line Ntera2, and primary human fetal neurons [54]. In addition, EBV has been shown to infect HMC-3, a microglial cell line, and U-87 MG, an astrocyte cell line [55].

This review will begin with an overview of EBV reactivation and the lytic and latent cycles, including recent advances in our understanding of how EBV establishes and maintains latency. It will next explore how cellular and psychological stressors lead to EBV reactivation. We will conclude with a brief overview of advances in treatments targeting EBV.

## 2. Overview of EBV Reactivation and the Lytic Cycle

Herpesvirus lytic replication involves three stages of gene expression: immediate early, early, and late [56]. The transcription factors BZLF1, also known as Zta, ZEBRA, EB1, or Z, and BRLF1, also known as Rta or R, are critical to the reactivation of the lytic cycle. As the master regulator, BZLF1 is particularly important in this activation [57]. These two genes induce the other’s expression [58]; in fact, one key role of ZEBRA, the protein encoded by BZLF1, is to stimulate the BRLF1 gene, which leads to the production of the protein Rta [57]. Transcription from oriLyt requires BZLF1 and BRLF1 expression. Expression of the early gene BSMLF1, also known as SM, Mta, and EB2, is also essential in gene transcription. The protein encoded by this gene has been shown to both upregulate lytic gene synthesis and downregulate host protein synthesis by impacting mRNA stability and transport through a direct interaction with the RNA [59,60]. BMRF1 is another key early protein that interacts with the BALF 5 DNA polymerase subunit to enhance nucleotide processivity. It does this by stabilizing the interaction between the primer template and the polymerase [61]. Early genes have diverse functional roles outside of replication as well. For example, BARF1 is an early gene involved in immune modulation. Specifically, it was found to inhibit colony-stimulating factor-1 activity through mimicry of the colony-stimulating factor-1 receptor c-fms [62]. BHRF2 is another EBV early gene that exerts its effect through molecular mimicry. It was shown to resemble the human antiapoptotic protein Bcl-2. Like Bcl-2, it can improve B cell survival [63].

Requirements for late gene viral transcription include viral DNA replication [64,65] and the interaction between the EBV-encoded BCRF1 protein and a viral pentamer with cellular RNA polymerase II [66]. The late phase of the lytic cycle involves the production of structural proteins and virion assembly. Examples of late genes include genes coding for structural proteins (such as BcLF1 and BNRF1), glycoproteins (such as BLLF1 and BXLF2), and viral interleukin-10 (BCRF1) [65].

## 3. Overview of EBV Latency

**Figure 1:**
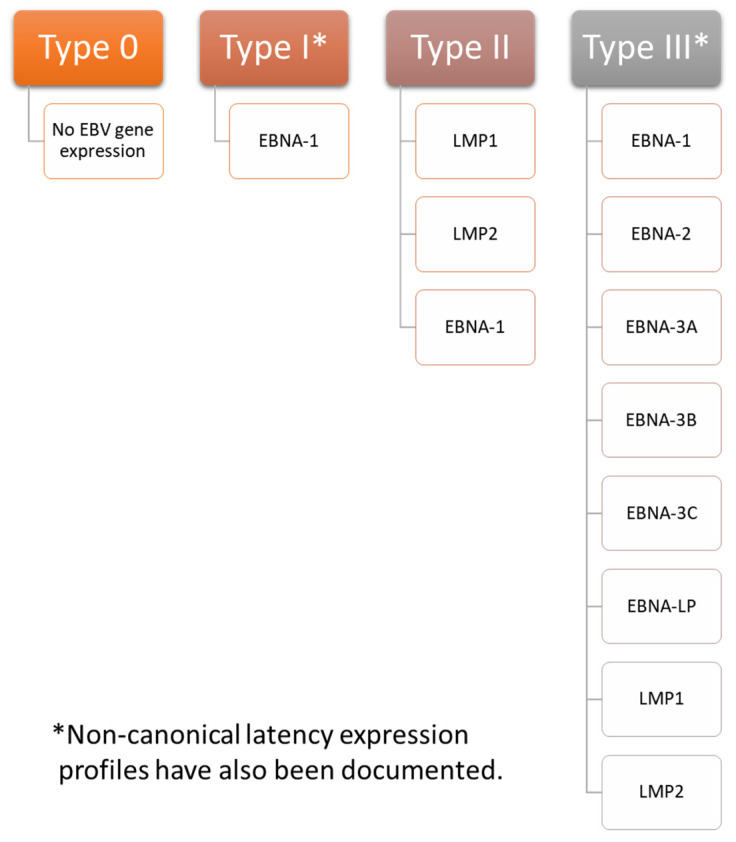
EBV Latency Types. EBV has four gene expression profiles during latent infection: type 0, type I, type II, and type III. In type 0 latency EBV, few, if any, proteins are expressed. Epstein-Barr nuclear antigen (EBNA)-1 is the only protein expressed during type I latency. Latent membrane protein (LMP) 1, LMP2, and EBNA-1 are all expressed during type II latency. All genes associated with latency are expressed in type III latency, including EBNA-1, EBNA-2, EBNA-3A, EBNA-3B, EBNA-3C, EBNA-LP (leader protein), LMP1, and LMP2. These proteins play important roles in maintaining latency. Notably, non-canonical latency expression profiles have also been documented. For example, infected cells expressing a type I latency profile may also express LMP1 or LMP2A, and infected cells expressing a type III latency profile may not express EBNA-2.

Latent infections can establish one of four programs of gene expression: type 0, type I, type II, or type III (summarized in Figure 1). The proteins expressed play important roles in maintaining latency. A number of factors determining which latency program is ultimately expressed have been identified. For example, expression from the C promoter (cP) is essential in establishing type III latency. In type I latency, the cP is silent while the Q promoter (Qp) becomes active. An analysis of the three-dimensional structure of chromatin showed that the latent origin of replication (oriP), a section of the EBV chromosome important in replication and plasmid maintenance [67], is found near cP during type III latency, but near Qp during type I latency. CCCTC-binding factor (CTCF), which is a zinc finger protein important in creating DNA loops [68], has been implicated in modulating the association between oriP and either cP or Qp [69]. Furthermore, poly(ADP-ribose) polymerase I (PARP1) stabilizes CTCF binding and ensures that chromatin remains open during type III latency [70]. Histone H3 and H4 acetylation provide an additional layer of control over cP and Qp activation [71]. Establishment of type II latency occurs in the presence of IL-10, which can stimulate LMP1 production without concomitant stimulation of EBNA-2. This indicates that it may play a role in the establishment of type II latency [72]. EBV-infected cells expressing other types of latency can be converted to type II latency by IL-21, which stimulates latent membrane protein (LMP) 1 but not Epstein-Barr nuclear antigen (EBNA)-2 in type I latency. It inhibits cP and LMP2A mRNA while upregulating LMP1 mRNA in type III latency [73]. Type 0 latency is seen in B cells that are not undergoing active division [35].

Few, if any, proteins are expressed during type 0 latency. EBNA-1 is the primary viral gene expressed in type I latency, although other latency-associated proteins are occasionally expressed as well. Infected cells exhibiting a type II latency pattern express LMP1 and 2 in addition to EBNA-1. Infected cells with a type III expression profile express all latency associated genes, including EBNA-1, EBNA-2, EBNA-3A, EBNA-3B, EBNA-3C, EBNA-LP (leader protein), LMP1, and LMP2 [35,74,75]. Of note, non-canonical patterns of EBV expression exist. For example, cells exhibiting a type I latency program may also express LMP1 or LMP2A [76], and cells with a type III latency expression profile may not express EBNA-2 [77].

Type I latency is associated with Burkitt lymphoma and can be modeled in Akata, Mutu, AG876, GC1, and YCCEL1 EBV strains. Type II latency can be found in nasopharyngeal carcinoma, Hodgkin lymphoma associated with EBV, and T cell lymphoma and can be modeled with the C666-1 EBV strain. Type III latency is seen in lymphoblastoid cell lines. It can be modeled with the B95-8, Raji, GD1, and GD2 EBV strains [35,74].

EBNA-1 is expressed in every lytic expression program except type 0. It contributes to the stability of the latent EBV episome [78], which is the EBV genome conformation most commonly assumed during latency [79], by playing roles in episomal replication and mitotic segregation. EBNA-1 activates other latent genes that lead to B cell immortalization [78] and tethers the viral episome to the host genome [80]. EBNA-2 is a transcription factor that interacts with DNA through the adaptor proteins, C-promoter binding factor (CBF) 1 and PU.1. It stimulates the C promoter as well as the promoters for LMP1, LMP2A, and LMP2B [81]. Of note, the C promoter is important in EBNA gene expression [82]. EBNA-2 is also essential for B cell immortalization [83]. The best characterized function of EBNA-LP is as a coactivator of EBNA-2 [81,84], but it has also been implicated in modulating other cellular and viral functions, such as apoptosis and cell survival [85,86]. It has been shown to help recruit transcription factors to the viral genome [86]. The EBNA-3 proteins are transcription regulators, with EBNA-3A and EBNA-3C acting as oncogenes and EBNA-3B acting as a tumor suppressor [87]. While EBNA-3B is nonessential for B cell immortalization [88], EBNA-3A and EBNA-3C are essential [89]. LMP1 is best known for its role in B cell immortalization, but it is also involved in a myriad of other viral functions, such as cell contact/migration, immunomodulation, changes in gene and miRNA expression, and stimulation of tumor invasion [90]. LMP2 has two isoforms, LMP2A and LMP2B. Together, these proteins are involved in transformation, proliferation, migration, and latency [91]. The two isoforms have conflicting roles in B cell receptor signaling, with LMP2A inhibiting B cell receptor signal transduction when expressed alone. Co-expression with LMP2B restored normal signal transduction [92]. LMP2A is also essential in generating surrogate cell survival signals [76,93].

In addition to the above proteins, EBV generates a host of RNAs during latency, including BamHI fragment A rightward transcripts (BARTs) and Epstein-Barr virus-encoded small RNAs (EBER). These RNAs play many essential roles in latently infected cells, including but not limited to contributions to cell survival, immunoevasion, cell proliferation, immunomodulation, malignant transformation, and maintaining viral latency [94,95,96,97,98]. Interestingly, EBER deletions have been shown to have no effect on establishing transformed lymphoblastoid cell lines [99], and it has been proposed that they act as a backup for LMP1 [100]. By contrast, BARTs have been shown to play a role in latency [101,102].

### Establishment and Maintenance of Latency in EBV Infection

B cell immortalization is strongly associated with latency [103]. EBV has been shown to stimulate reactive oxygen species (ROS) production in cells [104,105]. This oxidative stress is required to immortalize B-cells. In fact, Chen, Kamranvar, and Masucci showed that the addition of ROS scavengers, such as N-acetylcysteine amide (NACA) and reduced glutathione, to infected B cells significantly inhibited their proliferation. No such adverse effect on cell growth was noted when ROS scavengers were added to mitogen-stimulated B cells. The authors further found that ROS production was necessary for normal expression of LMP1 and that ROS scavengers decreased signal transducer and activator of transcription 3 (STAT3) phosphorylation [106]. B cells derived from patients with a STAT3 negative mutation have previously been shown to resist EBV-induced immortalization, indicating the importance of STAT3 phosphorylation and activation in B cell transformation [107].

The early lytic cycle gene BHLF1 was recently shown to play a role in establishing latent infection and in B cell immortalization. Importantly, BHLF1 protein expression required the presence of the post-transcriptional regulator protein SM, which is only expressed during the lytic cycle. This requirement indicates that the latency-associated functions of BHLF1 are mediated by long noncoding RNA (lncRNA) rather than by the protein itself. While BL2 cells infected with both wild-type EBV and EBV carrying deletions of the BHLF1 open reading frame (ORF) and 5′ promoter region both established type III latency, cells infected with the mutant strain transitioned to a type I latency program by two months post infection. Cells infected by wild-type virus continued to express type III latency genes. This trend was also observed with deletions targeting the BHLF1 ORF alone, although the cells infected with this strain remained in type III latency for slightly longer than those infected with EBV containing deletions to both the BHLF1 ORF and 5′ promoter region. In addition, B cells from three out of four donors infected with either deletion demonstrated impaired B cell immortalization when compared to wild-type EBV. Cells from one donor were transformed equally effectively regardless of which of the three viruses were used to infect the cells, although the authors noted that these B cells appeared to be more sensitive to transformation than B cells from other donors [108]. Notably, the above experiments on B cell transformation were performed in vitro. There may be additional factors involved in in vivo transformation.

CRISPR screening recently identified MYC, an important cellular protein with many functions (reviewed in [109]), as a key regulator of EBV latency. Induction of lytic infection in Burkitt lymphoma cells was correlated with decreased MYC expression. MYC depletion resulted in increased expression of BZLF1 and BMRF1 in Akata and P3HR-1 cells, while eliminating MYC caused an increase in the expression of the late protein gp350 in Burkitt lymphoma cells. RNA sequencing analysis demonstrated that MYC depletion resulted in the upregulation of 77 genes associated with the EBV lytic replication cycle. There was a corresponding increase in the EBV genome copy number. MYC knockdown in lymphoblastoid B cells, which have a type III latent gene expression profile, also induced BZLF1 and BMRF1 expression. BMRF1 was not as strongly stimulated in lymphoblastoid cells, which the authors postulated may have been due to tet methylcytosine dioxygenase 2 (TET2) demethylase-mediated inhibition of BZLF’s ability to stimulate early gene expression. gp350 expression was abrogated in BZLF1 knockout cells, indicating that MYC acts on BZLF1 to promote latency. Subsequent experiments indicated that MYC acts on the BZLF1 promoter and interacts with EBV DNA near both oriLyts (origin of lytic replication). Chromatin conformation capture (3C) assay showed interactions between the BZLF1 promoter and the oriLyt T6 R primer as well as interactions between BZLF1 and the T10 primer of the TR region. These interactions did not occur in the presence of MYC overexpression. The authors proposed that loss of MYC results in oriLyt and TR-DNA looping to the BZLF1 promoter to trigger lytic replication [110].

The same set of experiments demonstrated that factors associated with MYC influence EBV latency. For example, depleting the cohesin structural maintenance of chromosomes 1A (SMC1A) also stimulated 77 EBV genes associated with the lytic cycle and increased the EBV genome copy number. Consistent with these results, MYC mRNA expression was significantly decreased. Likewise, the facilitated chromatin transcription (FACT) complex, which previously was shown to interact with MYC [111], impacts MYC expression. CRISPR targeting of SUPT16H, a FACT subunit, reduced MYC expression by 65% and stimulated 67 lytic genes. Additionally, SUPT16H and SSRP1, another FACT subunit, are both upregulated by EBV during initial infection. Other proteins in which the CRISPR knockout induced lytic gene expression included STAGA, GCN5 histone acetyltransferase/lysine acetyltransferase 2B (PCAF), and Mediator, which is recruited by STAGA and MYC [110].

EBV is capable of manipulating C-X-C motif chemokine receptor 4 (CXCR4) expression to maintain latency in EBV-related gastric carcinoma. EBV was shown to increase CXCR4 expression by activating the AKT/PI3K pathway. Notably, LMP2A was found to increase CXCR4 expression, and BZLF1 expression increased in AGS cells treated with siRNA targeting CXCR4. CXCR4 decreased the viral copy number and stimulated LMP2A and EBNA-1. These results indicate that CXCR4 is necessary for EBV latency [112].

Small ubiquitin-related modifier (SUMO) modification is a reversible physiologic process involved in regulating transcription, remodeling chromatin, and responding to hypoxic stress [113,114]. Like so many other cellular processes, it has been subverted for use by EBV [113]. EBV has three separate potential SUMO interaction motifs (SIMs) called SIM1, SIM2, and SIM3, of which SIM2 and SIM3 were shown to be important in EBNA-1’s ability to bind to His-tagged SUMO1 and SUMO2 proteins. SIM2 deletion inhibited EBNA-1 dimerization, while SIM3 deletion inhibited poly-SUMO2 modification of EBNA-1. Chromatin immunoprecipitation (ChIP) assays demonstrated that mutations in both SIM2 and SIM3 impaired the binding of EBNA-1 to oriP chromosomal DNA. Cells infected with EBV containing SIM3 or K477R (a SUMOylation site) mutations as well as SIM2, SIM3, and EBNA-1 with glycine/alanine deletions demonstrated impaired oriP mini genome maintenance. Furthermore, SIM3 deletion and K477R mutation stimulated the transcription of BZLF1, an immediate early protein important in lytic gene expression [115], while deletions in SIM2 or SIM3 caused increased transcriptional activity of the BZLF1 promoter. Immunoblot analysis demonstrated that SUMO1-modified proteins increased in frequency in the presence of EBNA-1, while SUMO2-modified proteins decreased in frequency. Notably, SUMO2-associated proteins targeted by EBNA-1 were primarily associated with the proteasome regulatory complex and the ubiquitin-dependent Cullin-RING E3 ligase. This indicates that EBNA-1 may target proteins with SUMO2 modifications for degradation. Pathways affected include those involved in the viral life cycle, gene transcription, gene expression, and mRNA metabolic processes. Those affecting DNA and RNA binding were particularly prominent in affected mRNA metabolic process pathways. In addition, proteins associated with EBNA-1 SIM sites were mainly associated with DNA and RNA binding, gene transcription and expression, and other proteins involved in proteasome-mediated degradation. Lastly, the authors showed that hypoxia-induced EBV reactivation stimulated an increase in SUMO1-modified STIP1 homology and U-box containing protein 1 (STUB1) and tripartite motif containing 28 (KAP1), while the SUMO2 modified forms decreased. Hypoxia stimulated the preferential association of EBNA-1 with SUMO2 over SUMO1. It also downregulated the association between EBNA-1 and SUMO2-modified KAP1, which, along with ubiquitin specific peptidase 7 (USP7), make up a SUMO2-modified complex. STUB1 inhibition decreased the EBV genome copy number, while USP7 knockdown had the opposite effect. KAP1, USP7, and STUB1 knockdown cells exposed to hypoxic conditions had higher BZLF1 levels than the control [116].

Paired box (PAX) 5 is a cellular oncogene involved in activating genes that promote differentiation into B cells and in inhibiting genes that promote differentiation into other cell types [117]. More recently, it has been shown to physically interact with EBNA-1. Expression of a short hairpin RNA targeting the 3′ untranslated region of PAX5 nearly eliminated the activity of the oriP-Luc reporter plasmid, which is an oriP-SV40-Luciferase expression vector dependent on EBNA-1 expression [118]. This defect was corrected in the presence of plasmid-generated Flag epitope-tagged PAX5 (FPAX5). PAX5 depletion resulted in a 70%–95% decrease in EBNA-1 enrichment at oriP and a 50%–95% decrease in nucleolin (NCL, an EBNA-1 associated protein) enrichment at TR-DNA, as measured by ChIP assays. This is consistent with a model in which PAX5 is necessary for EBNA-1 to localize to EBV oriP or TR-DNA. It was also shown that PAX5 associates with the transcription enhancers p300 and histone 3 lysine 4 trimethyl (H3K4me3). PAX5 knockdown resulted in the dissociation of p300 from oriP DNA as well as the dissociation of both p300 and H3K4me3 from TR-DNA. EBNA-1 dissociated from both oriP DNA and TR-DNA. Lastly, the authors showed that PAX5 knockdown reduced the EBV genome copy number, a finding that was reversed in the presence of transfected FPAX5 [119].

Subverting histone chaperone proteins is another mechanism by which EBV maintains latency. For example, the chromatin assembly factor (CAF) 1 complex supplies histone 3 and histone 4 dimers to replication forks. Depletion of any of CAF’s three subunits (CHAF1A, CHAF1B, and RBBP4) induced the expression of BZLF1 (an immediate early gene) and BMRF1 (an early gene) as well as numerous transcripts associated with lytic infection. These results were seen in multiple Burkitt’s lymphoma cell lines. In addition, it has been shown that inhibiting EBNA-2 resulted in downregulation of CAF1 subunit mRNA expression. Downregulating histone loader histone regulatory homologue A (HIRA), which loads H3.3 and H4 complexes onto DNA, had a similar result in that it increased BZLF1 and BMRF1 expression. Unlike CHAF1A and CHAF1B, there was no decrease in HIRA mRNA levels. Additionally, targeting alpha thalassemia/mental retardation syndrome X-linked chromatin remodeler (ATRX) and death domain-associated protein (DAXX), which load histone 3.3, with CRISPR stimulated BZLF1 and BMRF1 expression. This indicates that EBV subverts multiple histone loaders to maintain latent infection. Interestingly, reduction in CHAF1B levels decreased the concentration of histone 3.1 and histone 3.3 at the promoters for BZLF1 and BLLF1, and CHAF1B single guide RNA (sgCHAF1B) reduced histone 3.1 presence at oriLyt L and oriLyt R, which are important in initiating lytic gene expression. Levels of histone 3.1 and histone 3.3 were both increased by two days post infection, indicating that these histones are transferred to incoming EBV episomes. The same study showed that CHAF1B knockdown decreased histone 3 lysine 9 trimethyl (H3K9me3) at the promoters for BZLF1, BLLF1, oriLyt R, and oriLyt L, and that sgCHAF1B reduced histone 3 lysine 27 trimethyl (H3K27me3) occupancy at these sites in Akata cells [120]. Both H3K9me3 and H3K27me3 are repressive histone modifications [121].

Recent research has demonstrated the importance of miRNA in maintaining latency. Screening identified eight miRNAs (BHRF1-2, BART1, BART2, BART8, BART11, BART18, BART9, and BART17) whose expression diminished nuclear factor kappa B (NF-κB) expression, which was used as a proxy for B cell receptor activation. In addition, BHRF1-1, BHRF1-2, BART14, and BART18 expression significantly reduced AP-1 signaling. B cell receptor engagement leads to the activation of the transcription factor AP-1 via the GTPase Ral [122]. Furthermore, many targets of EBV mRNAs are involved in B cell receptor signaling, such as growth factor receptor-bound protein 2 (GRB2), SOS Ras/Rac guanine nucleotide exchange factor 1 (SOS1), Ras-related C3 botulinum toxin substrate 1 (RAC1), and Ikk-B. shRNA targeting GRB2, SOS1, or RAC1 resulted in a sharp downregulation of NF-κB. Intriguingly, miR-BHRF1-2-5p targets all three of these proteins. Indeed, miR-BHRF1-2-5P inhibition stunted the growth of both lymphoblastoid and EBV-associated diffuse large B cell lymphoma cell lines [123]. A summary of mechanisms involved in EBV latency can be found in Table 1.

## 4. Factors Involved in EBV Reactivation

EBV reactivation and cell differentiation are closely associated. Crawford and Ando found that PC1, an antigen indicating B cell maturation, was observed in lytically infected cells and that the induction of B cell differentiation after treatment with TPA increased expression of the viral capsid antigen (VCA), which is associated with lytic replication [124]. It was later shown that the promoter for BZLF1 only becomes active after B cell differentiation into plasma cells [125].

Similar trends have been noted in epithelial cells. For example, Nawander et al. demonstrated that EBV lytic replication was restricted to the more differentiated cell sections in an organotypic raft culture. Furthermore, they showed that Krüppel-like factor 4 (KLF4), a cellular factor that has previously been shown to promote cell differentiation [126], binds to and activates the BZLF1 and BRLF1 promoters [127].

B-lymphocyte-induced maturation protein 1 (BLIMP1), another cellular transcription factor implicated in cell differentiation, was also shown to induce lytic reactivation in numerous examined cell lines, including all epithelial cell lines and a portion of B cell lines, by stimulating transcription from both the BZLF1 and BRLF1 promoters [128]. In addition, BLIMP1 was found to act synergistically with KLF4 to enhance EBV lytic reactivation [127]. BLIMP1 and KLF4 stimulate LMP1 production by activating LMP1 promoters. Of note, neither BZLF1 nor BRLF1 were required for LMP1 induction. Following LMP1 activation, KLF4, BLIMP1, and LMP1 then work in conjunction to activate BZLF1 and BRLF1 expression [129].

**Figure 2:**
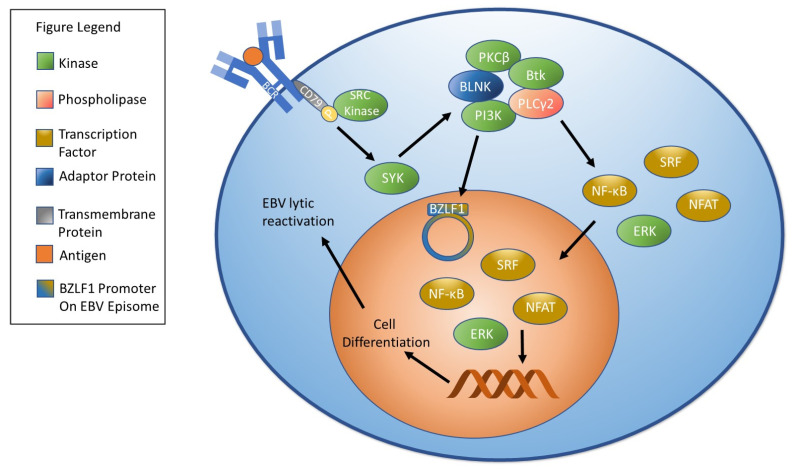
B Cell Receptor Signaling and EBV Reactivation. B cell receptor signaling begins when an antigen binds to the B cell receptor. Binding causes SRC kinases to phosphorylate CD 79, which stimulates SYK. SYK stimulation results in the formation of a signalosome comprised of PI3K, Btk, BLNK, PLCγ2, and PKCβ. Signalosome activation results in the activation of molecules that influence gene expression including NF-κB, NFAT, ERK, and SRF. Stimulation of the B cell receptor leads to plasma cell differentiation and can trigger EBV reactivation. Notably, activation of PI3K has been shown to stimulate EBV reactivation.

Cross-linking of cell surface immunoglobulins was first identified as a key stimulant for EBV reactivation when it was shown that anti-human immunoglobulin antibodies stimulated lytic replication when cells express the appropriate immunoglobulin chain [130]. In brief, B cell activation begins when antigen binding stimulates phosphorylation of CD79 immunoreceptor tyrosine-based activation motifs (ITAMs) by SRC kinases including LYN, FYN, and B-lymphoid tyrosine kinase (BLK). In turn, this activates spleen tyrosine kinase (SYK). SYK activation stimulates the formation of a signalosome that includes signaling molecules such as PI3K, Bruton’s tyrosine kinase (Btk), the adaptor protein [131] B cell linker protein (BLNK), phospholipase Cγ2 (PLCγ2), and protein kinase C (PKC) β [132,133]. Activation of the signalosome sets in motion signaling cascades that activate downstream molecules including the transcription factors [134,135,136] nuclear factor of activated T cells (NFAT), NF-κB, and serum response factor (SRF) as well as the kinase [136] extracellular signal-related kinase (ERK) [132,137]. One consequence of B cell receptor (BCR) activation is differentiation into plasma cells [138]. As discussed above, terminal differentiation can stimulate EBV reactivation [125]. A brief overview of B cell receptor signaling can be found in Figure 2.

Several aspects of BCR-mediated EBV activation have been elucidated. For example, PI3K signaling has been shown to play a role in EBV activation through the B cell receptor. Of the six cell lines studied (Akata, Daudi, Mutu-I, Sav-I, Kem-I, and Oku-I), anti-Ig treatment stimulated EBV reactivation in the majority of Akata cells and some Mutu-I and Sav-1 cells, as measured by expression of the early protein BHRF1. It did not induce reactivation in Daudi, Kem-I, or Oku-I cells. Interestingly, AKT and ERK proteins were phosphorylated in Akata and Mutu-1 cells following anti-Ig treatment. By contrast, AKT phosphorylation was absent in Daudi cells and ERK phosphorylation was limited. Stimulation of the PI3K pathway in Daudi cells with IGF-1 and treatment with anti-Ig resulted in EBV reactivation in 12% of cells, as measured by BHRF1 expression. BZLF1 was also detected in cells harboring active EBV. IGF-1 and anti-Ig treatment also increased phosphorylation of ERK and p38, a MAP kinase, in Daudi cells. In addition, transfection of the BZLF1 promoter-luciferase gene plasmid in EBV-negative Daudi cells and subsequent treatment with IGF-1 and anti-Ig resulted in activation of the BZLF1 promoter. This indicates that BCR signaling targets the BZLF1 promoter and that PI3K signaling plays a role in BZLF1 activation by BCR stimulation. [139]. Both this study and others have indicated that PI3K inhibition is detrimental to EBV reactivation through the BCR [139,140]. Other protein kinases in which inhibition has been shown to reduce the genome copy number following BCR activation include PKC, glycogen synthase kinase 3β (GSK-3β), platelet-derived growth factor receptor-associated tyrosine kinase (PDGFRK), and epidermal growth factor receptor-associated tyrosine kinase (EGFRK). Inhibition of the MAPK and NF-κB pathways was shown to have a similar effect [140].

Cellular interferon regulatory factor (IRF) 8 was recently shown to be important in lytic cycle induction via the BCR. IRF8 knockdown inhibits lytic replication, as measured by levels of the lytic proteins BZLF1 and BGLF4. In addition, viral copy numbers and RNA levels of BZLF1, BRLF1, and the late gene BGLF2 were inhibited in IRF8 knockdown cells. RNA-Seq followed by gene ontology and manual curation revealed that genes involved in facilitating apoptosis were downregulated in IRF8-depleted cells. This led the authors to show that PARP and caspase cleavage was reduced in IRF8 knockdown cells, indicating IRF8 depletion inhibits apoptosis following BCR-mediated induction of the lytic cycle. Consistent with these results, levels of caspase-3 and caspase-8 decreased in IRF8 knockdown cells, although levels of caspases-2, -7, and -9 were less affected. Caspase gene expression was unchanged except for caspase-1. In addition, levels of the antiapoptotic protein BCL-2 increased in IRF8-depleted cells. Strikingly, caspase inhibition significantly decreased expression of the immediate-early genes BZLF1 and BRLF1, the early gene BGLF4, and the late gene BGLF2. Additionally, the authors showed that expression of KAP1, PAX5, and DNA methyltransferase 3 alpha (DNMT3A), all genes involved in latency, demonstrated decreased suppression in IRF8 knockdowns. While their expression was decreased during lytic activation, infected Akata cells pretreated with a caspase inhibitor demonstrated normal expression of KAP1, PAX5, and DNMT3A following BCR-mediated lytic activation. In essence, caspase activation destabilizes cellular restriction factors and promotes EBV activation [141].

The authors next evaluated the role of caspases in EBV reactivation. Caspase-1, the promoter of which was shown to be regulated by both IRF8 and IRF1 in this set of experiments, was depleted. Depletion decreased the viral copy number and reduced expression of BZLF1, BRLF1, and BGLF2. Mechanistically, it was determined that caspase-1 stimulates EBV reactivation in part by cleaving KAP1. Depleting both KAP1 and caspase-1 stimulated EBV reactivation, cementing the role of KAP1 targeting by caspase-1 in reactivation [141].

### 4.1. Oxidative Stress/Reactive Oxygen Species and EBV Reactivation

Recent research has examined the role of oxidative stress in EBV reactivation. Chaetocin is an antiproliferative made by Chaetomium fungi that is capable of generating ROS [142]. Real-time RT-PCR revealed that chaetocin treatment upregulated the immediate early genes BZLF1 and BRLF1, the early gene BMRF1, and the late gene BLLF1. This was associated with an increase in the viral copy number. Treatment with the ROS inhibitor N-acetyl-L-cysteine counteracted the effect of chaetocin, indicating that ROS induction is associated with the lytic EBV cycle [143].

Hu et al. recently assessed oxidative stress levels in the setting of nasopharyngeal carcinoma. EBV-infected cells demonstrated higher levels of ROS as well as increased ratios of the redox pairs NADP+/NADPH and oxidized glutathione/total glutathione (GSSG/GSH), indicating that EBV does cause oxidative stress. Mechanistically, it was determined that EBV induced upregulation of NADPH oxidase (NOX) genes, which in turn contributed to increased levels of ROS. NOX inhibition decreased ROS levels. In addition, EBV infected cells upregulated nuclear factor erythroid 2-related-factor 2 (Nrf2), a transcriptional regulator important in the response to oxidative stress [144], and its target genes. A similar upregulation of both ROS and antioxidant pathways is seen in cancer cells; this dual upregulation allows the cells to carry out necessary cell functions in the setting of elevated ROS levels [145]. The authors postulated that this allows cells to undergo ‘redox resetting’, a process in which the cells are able to tolerate increasingly elevated ROS levels [146].

Hu et al. further showed that ROS induction can stimulate EBV reactivation. Treating infected nasopharyngeal carcinoma cells with H_2_O_2_, which increases ROS production, stimulated expression of EBV lytic proteins. Of note, they showed that LMP1 upregulates ROS production. LMP1 depletion decreased EBV lytic reactivation. This deficit was rescued after LMP1-deficient cells were treated with H_2_O_2._ In essence, LMP1 promotes EBV lytic reactivation through the generation of ROS [146].

The interaction between NRF2 and EBV was recently further elucidated by Yun, Kim, and Hur. They showed that siRNA targeting either LMP1 or LMP2A caused NRF2 concentrations to decrease and inhibited its translocation to the nucleus [147]. NRF2 translocation only occurs under conditions of oxidative stress. Once in the nucleus, NRF2 dimerizes with Maf proteins and recognizes enhancer sequences associated with NRF2 target genes. It then stimulates antioxidant and metabolic gene expression [144]. AKT inhibition resulted in decreased expression and nuclear translocation of NRF2. In addition, heme oxygenase-1 (HO-1) and NAD(P)H-quinone oxidoreductase 1 (NQO-1), which are targets of NRF2, were downregulated upon AKT inhibition, and inhibition of LMP1 or LMP2A downregulated AKT activation. This implies that AKT is also involved in LMP-mediated NRF2 signaling. The importance of NRF2 activation in EBV-infected cells is highlighted by the finding that infected cells underwent apoptosis in the presence of siRNA targeting NRF2 [147].

Working with lymphoma cell lines, Cao et al. recently elucidated the role of the cellular miRNA-18a in EBV reactivation. miRNA-18a transfection stimulated cell proliferation in EBV-positive P3HR-1 and Raji cells as well as EBV-infected BJAB cells by promoting the transition from G1 phase to S phase. miRNA-18a had no effect on the growth of uninfected cells. Additionally, transfecting miRNA-18a mimics in P3HR-1 and Raji cells increased the viral load and stimulated gene expression, including BZLF1. Cellular expression of miRNA-18a in cells infected with EBV increased following DNA damage secondary to UV radiation and hypoxia. In addition, hypoxia and radiation both increased the EBV viral load. Mechanistically, miRNA-18a was shown to target ATM, which is important in DNA damage repair. Hypoxic cells transfected with ATM reversed the effects of miRNA-18a and inhibited EBV gene expression. This contrasts with the effects of ATM transfection in normoxic conditions, where it induced lytic gene expression and inhibited latent gene expression [148].

Interestingly, a recent study showed that patients with oropharyngeal cancer had lower overall levels of antioxidants as well as the free radical scavengers glutathione peroxidase and superoxide dismutase. EBV+ carcinomas consistently had the lowest levels when compared to both control and EBV– carcinomas. In addition, cells bearing infections with wild-type LMP1 had lower levels of antioxidants, glutathione peroxidase, and superoxide dismutase than those infected by EBV with a deletion in LMP1. Additionally, the three tested measures decreased as EBV-specific antibody levels increased [149].

### 4.2. Co-Infection/Immunosuppression and Reactivation

Viral reactivation has long been an issue in the setting of immunosuppression or other immune stressors, and EBV is no exception. Post-transplant lymphoproliferative disorder (PTLD) is one such complication associated with EBV reactivation. As its name indicates, PTLD is a malignant B cell lymphoproliferation that occurs following transplantation and represents an important cause of post-transplant mortality [150].

Myalgic encephalomyelitis/chronic fatigue syndrome (ME/CFS) is another disease associated with EBV reactivation [151]. Symptoms include poor concentration, sleep disturbance, tender lymphadenopathy, musculoskeletal pain, pharyngitis, and severe fatigue [152].

γδ T cells are a subset of T cells in which the T cell receptor contains a γ-chain (TRG) and a δ-chain with roles in both innate and adaptive immunity [153]. Vδ2+ T cells are γδ T cells that have been shown to target EBV-infected cells [154], More recently, Vδ2+ T cell proliferation was shown to be adversely impacted by the immunosuppressant mycophenolate mofetil following transplant. Transplant patients were given either a shorter course (range of treatment duration 15–36 days, average 25 days) or a longer course (range 24–69 days, average 41 days) of mycophenolate mofetil. An analysis of T cell subsets showed that Vδ2+ T cells alone had consistently higher numbers at 60 days and 90 days post-transplant in the shorter duration group when compared to the longer duration group, indicating that Vδ2+ T cell recovery rates improve with a shorter duration of immunosuppression. The frequency of patients with EBV reactivation and EBV-related PTLD decreased from 26% and 10.6% to 13% and 2.4%, respectively. Treatment with the immunosuppressants mycophenolic acid and cyclosporin A eliminated Vδ2+ T expansion. In addition, there was a decrease in interferon-γ levels as well as in the expression of the receptors HLA-DR and NKG2D. Lastly, the authors showed that treatment with mycophenolate mofetil impaired the ability of Vδ2+ T cells to control PTLD tumors [155].

EBV can act as an opportunistic virus capable of taking advantage of host co-infection to reactivate. For example, a recent study assessed EBV reactivation in the setting of cytomegalovirus (CMV) and immunosuppressive or chemotherapeutic treatment. EBV DNA was detected in 52.7% of patients who also had detectable CMV DNA, while only 14.8% of patients without detectable CMV DNA had detectable EBV DNA. In the study, both age and CMV levels were associated with EBV reactivation. Interestingly, only 8 of the 15 patients in the sample greater than 75 years of age showed evidence of EBV reactivation, while all 11 patients aged 65–74 years old did. The authors postulated that this discrepancy may have to do with the relative intensities of treatment—older individuals may have received less intense therapy due to their age and may have had lower levels of immunosuppression as a result [156].

Syphilis is another disease that was recently shown to cause EBV reactivation. When T cell depleted, latently EBV-infected peripheral blood mononuclear cells (PBMCs) were stimulated with syphilis proteins, BZLF1 transcription increased, indicating EBV reactivation. The effect was particularly pronounced when the PBMCs were stimulated with both syphilis proteins and a BCR cross-linking antibody. Specifically, toll-like receptor (TLR) 2 stimulation in conjunction with BCR cross-linking resulted in a greater than 40-fold increase in EBV DNA. Stimulation of the other TLRs did not yield more than a minimal increase in EBV DNA. Stimulation of either TLR2 or the BCR resulted in changes in both TLR2 and BCR expression [157].

Likewise, human papillomavirus (HPV) can influence EBV reactivation. When the two viruses were co-expressed in NOK cells, an oral keratinocyte cell line, significantly more co-infected cells expressed BZLF1 and demonstrated EBV genome amplification than cells infected with only EBV. Mechanistically, it was shown that the HPV oncogenes E6 and E7 were responsible for HPV-mediated EBV lytic reactivation. Cells co-infected with E6 or E7 knockouts and EBV had similar rates of BZLF1 expression as cells infected with EBV only. Further experiments showed that no HPV proteins beyond E6 and E7 are required to stimulate EBV reactivation [158].

Interestingly, these results were not seen when attempting to establish EBV infection in keratinocytes already immortalized by HPV. When human tonsillar epithelial cells immortalized by HPV were infected with EBV, subsequent EBV levels were significantly lower in the HPV-infected cells than in HPV-negative cells. In addition, there was reduced expression of the immediate early genes BZLF1 and BRLF1 as well as the early genes BALF5 and BMRF1. While EBNA-1 and EBNA-2 did not show any changes in their expression profile, EBER1, an RNA associated with latency, did demonstrate increased expression. Interestingly, levels of BZLF1 were unchanged in human foreskin keratinocytes (HFK) in the presence of E6 and E7, which the authors attributed to the reduced differentiation potential of human tonsillar cells when compared to human foreskin keratinocyte cells. In a stark departure from the experiment discussed above, HFK and NOK cells expressing E6 and E7 contained lower EBV genome levels than the control. It was subsequently shown that while E6 had no effect on EBV replication levels, HFK cells expressing E7 had lower levels of EBV DNA. The authors demonstrated that decreased levels of KLF4 target genes may be responsible for the observed decrease in EBV replication [159]. KLF4 has been shown to augment EBV replication by activating the BZLF1 and BRLF1 promoters [127].

Kaposi sarcoma-associated herpesvirus (KSHV) is a gammaherpesvirus capable of establishing latent infection in B cells [160]. Given that EBV also establishes latent infection in B cells, it is unsurprising that they interact during co-infection. Indeed, infection with both KSHV and EBV resulted in a different gene expression profile than infection with EBV alone. For example, genes associated with mitochondrial function, the mitotic cell cycle, and apoptosis inhibition were upregulated while genes implicated in innate immunity and cytokine signaling were downregulated. These findings demonstrate that co-infection with KSHV may diminish innate immunity and activation of apoptotic processes while augmenting proliferation [161]. Gene expression in these co-infected cells was found to more closely resemble gene expression in primary effusion lymphoma cells (a B cell lymphoma associated with KSHV [162]) and plasma cells, including upregulation of proteins such as Aquaporin 3, BLIMP1, and IRF4. Furthermore, co-infection led to an increased transcription of lytic genes, including BZLF1, and decreased expression of latency associated genes, including EBNA1 and EBNA2. LMP1 and LMP2A expression was unaffected. Ultimately, the altered gene expression induced by KSHV co-infection, particularly the increased lytic gene activity, led to increased tumorigenesis in co-infected cells. Increased rates of lytic gene expression were noted in co-infected cells obtained from patients with lymphoproliferative disease [161].

Recent literature has examined the effect of the Coronavirus disease-19 (COVID-19) pandemic on EBV reactivation. EBV DNA was detectable in 28 of 34 patients admitted for COVID-19-associated respiratory failure, although nine patients did not have quantifiable EBV DNA levels. In addition, three patients were positive for EBV and CMV, four patients were positive for EBV and human herpesvirus (HHV)-6, and two patients were positive for EBV, CMV, and HHV-6. There was no association between EBV reactivation and mortality, although it was associated with a longer ICU stay [163].

EBV reactivation was not only seen in acute COVID infection. A separate study used EBV antibody titers to document EBV reactivation in 20 of 30 patients still experiencing COVID-related symptoms, including brain fog, fatigue, arthralgias, headaches, and others besides, at least 30 days after diagnosis. Only two of twenty controls, who had tested positive for COVID but were not experiencing lingering symptoms, were positive for EBV reactivation. This group had tested positive for COVID at least 90 days prior to the study; in a group of participants who tested positive 21–90 days prior to the study, six of nine patients with long COVID symptoms tested positive for EBV reactivation, while only one of eleven did in the control group. While the EBV antibody EBV EA-D IgG was correlated with experiencing long COVID symptoms, neither EBV VCA IgG antibody nor EBNA-1 IgG antibody demonstrated the same association [164].

### 4.3. Other Cellular Stressors and Reactivation

The integrated stress response (ISR) is a common cellular response to stressors that involves phosphorylating the eukaryotic translation initiation factor 2 alpha (eIF2α). eIF2α phosphorylation leads to a decrease in overall protein synthesis and an increase in the translation of certain genes involved in cell recovery and survival. eIF2α phosphorylation is mediated by the kinases heme-regulated eIF2α kinase (HRI), general control non-derepressible 2 (GCN2), PKR-like ER kinase (PERK), and double-stranded RNA-dependent protein kinase (PKR) [165]. 

A recent study examined the impact of the compound As_2_O_3_ on ISR stimulation and EBV reactivation. ISR induction was confirmed by the presence of elevated activating transcription factor 4 (ATF4), phosphorylated elF2α, and tribbles pseudokinase 3 (Trib3) RNA levels. Treatment with As_2_O_3_ stimulated BZLF1 transcription in time- and dose-dependent fashions in the BX1-Akata cell line. Targeting HRI with siRNA decreased expression of Trib3 and BZLF1. Additionally, lytic replication was blocked by HRI siRNA. While Trib3 and BZLF1 RNA levels increased when SNU719 (gastric carcinoma) and C666-1 (nasopharyngeal carcinoma) cells were treated with As_2_O_3_, there was only a minimal increase in BZLF1 protein in SNU719 cells and no detectable increase in C666-1 cells. Interestingly, BMRF1 RNA levels increased, but BMRF1 protein levels decreased in BX1-Akata cells. There was no evidence of gp350 production, but there was inhibition of virion production. Treatment with BTdCPU, which activates HRI, stimulated eIF2α phosphorylation, ATF4, and induced the lytic replication cycle. Treatment with both As_2_O_3_ and BTdCPU stimulated BZLF1 RNA production. Unlike As_2_O_3_, BTdCPU induced RNA expression of gp110 and gp350. Treatment with both drugs reversed this effect [166].

This conflicts with an earlier study showing that As_2_O_3_ treatment of Mutu cells inhibited lytic replication. This study found that BMRF1 was not induced in the presence of As_2_O_3_ and that BZLF1 and BRLF1 expression was lower in As_2_O_3_-treated cells than in non-treated cells. In addition to BZLF1, As_2_O_3_ inhibited expression of the lytic genes BMRF1, BGLF1 and VCA. As_2_O_3_ also was shown to block expression of LMP1 when given in conjunction with ganciclovir. Furthermore, As_2_O_3_ treatment decreased levels of EBV DNA in a variety of cell lines, including Mutu, JY, BX-1, and Akata cells. Moreover, As_2_O_3_ treatment decreased infected cell survival. Specifically, the authors found that As_2_O_3_ treatment inhibited EBV reactivation through SUMO1-induced ubiquitination of BZLF1 and its subsequent proteasomal degradation. Indeed, treatment with the proteasome inhibitor MG132 or the SUMOylation inhibitor ginkgolic acid restored BZLF1, BRLF1, and BMRF1 protein levels [167].

Radiation is another cellular stressor that increases levels of EBV reactivation. 2.0 Grays (Gy) of gamma radiation was shown to induce both BZLF1 and BLLF transcription in Akata cells with a peak at day 4, although the BZLF1 increase was not statistically significant due to a large standard deviation. It did become statistically significant by day 8. Additionally, doses as low as 0.1 Gy of radiation resulted in a significant increase in BLLF1 expression (but not BZLF1 expression) over the course of 16 days. High energy proton, carbon, and iron particles also increased BZLF1 and BLLF1 transcripts, and iron and carbon ions more efficiently induced intermediate early and late genes than gamma or proton radiation. All types of radiation also increased viral loads [168]. Table 2 contains a summary of reviewed factors capable of stimulating EBV reactivation.

### 4.4. Psychological Stressors

The effects of stress on the immune system have been extensively studied [169,170,171]. Thus, it is not surprising that psychological stress is associated with EBV reactivation. A recent study examined the effects of perceived stress on EBV antibody titers among women living in Appalachian Ohio. Appalachian women were selected due to their high levels of chronic stress. Perceived stress was assessed by questionnaire. A one point increase in perceived stress correlated with a 1.92% increase in the EBV antibody titer, while a one point increase in perceived social support correlated with a 1.00% decrease in the EBV antibody titer [172]. A similar study assessed how family social and economic instability between 0 and 5 years of age affected EBV viral DNA secretion as an adolescent. Moving into a new family household (for example, with a grandparent, new caregiver, or new parent) increased EBV shedding by 100% in those with evidence for prior EBV infection. EBV DNA shedding was also increased by experiencing at least one economic difficulty. There was no association between current family conditions and EBV DNA shedding [173].

A study analyzing the correlation between social capital and EBV antibody titers in Fujian, China demonstrated a linear correlation between the antibody titer and individual-level structural social capital, but an inverse correlation between the antibody titer and community-level structural capital [174]. This is in accordance with previous studies demonstrating lower levels of psychological stress in those with higher community social capital [175]. While there is conflicting literature regarding individual social capital and stress levels [175,176], the authors postulated that the obligations associated with social interactions may cause enough stress to outweigh the benefits associated with the interactions. The social interactions may lead to increased social regulation and more psychological stress [174].

Having family members move away has also been associated with elevated EBV titers. A recent study examining EBV antibody levels in participants living in rural Fujian, China, who had household members leave, demonstrated that there was a significant correlation between being left behind and EBV antibody titer. In addition, titer levels were higher when household members moved farther away. While there were slightly lower antibody titers in participants who were left behind because of marriage or education, the difference was not statistically significant. The study concluded that being left behind is associated with worsened psychological health and a subsequent increase in the EBV antibody titer [177].

Stress is closely related to pain [178,179]; thus, it is unsurprising that an association has been found between pain and EBV reactivation. Participants in a study assessing the relationship between pain and EBV reactivation found that older adults who reported more pain had increased antibody titers. Those who experienced more pain had a stronger association between age and the EBV titer than those who had less pain. In addition, age was associated with the EBV titer [180].

**Figure 3:**
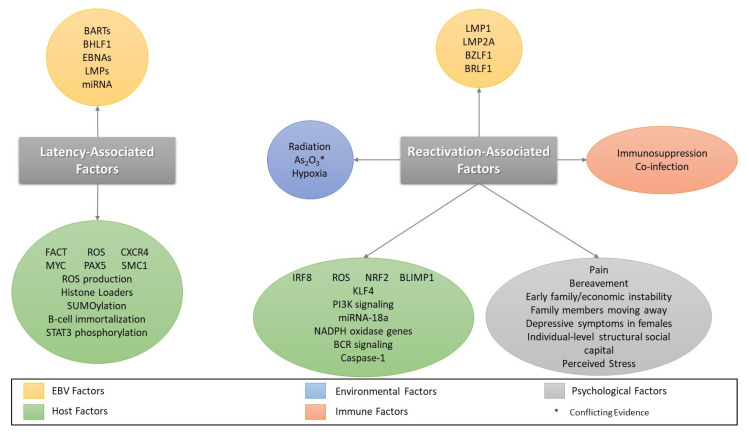
Summary of Factors Involved in Latency and Reactivation. Factors associated with latency and reactivation are categorized by source.

Interestingly, sex has been shown to play a role in EBV reactivation. For example, a recent study examined the effects of depressive symptoms on EBV reactivation in adolescents. While no correlation was noted between increasing depressive symptoms and EBV shedding in the general population or the male subpopulation, there was an increase in EBV shedding in the female subpopulation [181]. The authors postulated that this may be caused by differences in sex hormones and the stress response. For example, the authors pointed to reports that testosterone weakens the hypothalamic–pituitary–adrenal (HPA) stress axis response and that normal fluctuations in estrogen and progesterone related to the menstrual cycle impact cortisol responses [182]. They also noted that estrogen has been known to exacerbate B-cell diseases [183]. Differences between the sexes were also noted when assessing EBV antibody titers in response to spousal death. Males had lower EBV antibody titers than females. This finding was correlated with higher male inhibition, or the ability to control and divert attention away from emotion in the presence of stressors [184]. Figure 3 contains a summary of factors involved in latency and reactivation.

## 5. Advances in EBV Antiviral Therapy

Potent antivirals suitable for long-term prophylaxis has been recommended in other viruses capable of reactivation, such as the hepatitis B virus (HBV) [185]. While an antiviral prophylaxis approach is likewise warranted as an effective strategy for preventing EBV reactivation, no antivirals have been approved for use in EBV to date [186]. However, recent research has sought to address this deficiency. For example, Drosu et al. recently assessed the efficacy of the prodrugs tenofovir disoproxil fumarate (TDF) and tenofovir alafenamide (TAF), which are metabolized to the acyclic nucleoside/nucleotide analog tenofovir, in treating EBV reactivation. Both TDF and TAF were shown to reduce the number of viral copies in EBV+ HH514-16 cell lines by greater than 99.9% following stimulation of the lytic cycle by sodium butyrate. There was no treatment effect following TAF/TDF treatment of latently infected cells. Both TAF and TDF were shown to have lower half-maximal inhibitory concentration (IC_50_) values than penciclovir and acyclovir, while TAF also had a lower IC_50_ than ganciclovir. Further experiments focusing on TAF demonstrated inhibition of all six late lytic viral genes tested. Early lytic viral gene expression was only marginally affected. Specifically, it was shown that tenofovir diphosphate, the active form of the drugs, inhibits the EBV DNA polymerase via direct competition with dATP. While its ability to inhibit EBV DNA polymerase was more potent than acyclovir, it was less potent than ganciclovir. Interestingly, pretreatment with TAF was also shown to inhibit EBV lytic reactivation [21].

Dipyridamole is another drug whose efficacy was evaluated as a therapeutic option for EBV. Akata cells exposed to dipyridamole demonstrated dose-dependent inhibition of virion production following antibody-stimulated reactivation. A similar effect, although of lesser magnitude, was noted in B95-8 cells. While no effect was noted on latently infected cells, dipyridamole significantly impaired EBV viral replication in lytically infected Akata and B95-8 cells. Transcription of both immediate early and early genes were significantly inhibited in multiple cell lines. Notably, inhibition was reversed when cells were exposed to dipyridamole in the presence of excess adenosine or thymidine, indicating that dipyridamole exerts its antiviral effects through nucleoside transport inhibition [187].

Proton pump inhibitors (prazole compounds) are best known for their role in treating gastroesophageal reflux disease [188]. Recent literature has suggested they may have antiviral capabilities as well. For example, it was shown that tenatoprazole impaired the release of viral particles in a dose-dependent manner. There was a corresponding increase in the number of intracellular viral particles. This indicates that tenatoprazole interferes with viral particle egress, potentially through interfering with interactions between the Tsg101 and ubiquitin [189]. Tsg101 is involved in the endosomal sorting complex required for transport (ESCRT) process (reviewed in [190]), which has been implicated in EBV egress from infected cells [191]. Subsequent research demonstrated that numerous prazoles, including tenatoprazole, ilaprazole, and rabeprazole, significantly decreased detectable viral particles in the supernatant and on the surface of lytically infected cells. Consistent with the idea that they inhibit viral egress, treatment with prazoles did not appear to influence lytic gene expression. Electron microscopy revealed changes in the appearance of viral capsids in the presence of prazole compounds, including an increase in the frequency of defective/empty capsids and changes in virion size. In sum, prazoles interfere with both nuclear capsid maturation and exocytosis. In addition, mutation of cysteine 73 in the ubiquitin E2 variant (UEV) domain to alanine, which nullifies the effect of prazole treatment without altering protein function, rescued viral production in infected cells. The authors concluded that prazoles inhibit ubiquitin’s ability to bind to Tsg101 at cysteine 73, which in turn prevents EBV capsids from exiting the nucleus [192].

### 5.1. Advances in EBV Vaccines

Vaccination is an attractive strategy to manage viral reactivation. Indeed, clinical trials have examined the efficacy of vaccination in preventing reactivation in multiple viruses, including HBV [193,194], varicella zoster [195], and cytomegalovirus [196]. Indeed, the varicella zoster vaccine is in clinical use today [195]. Recent work in EBV vaccination technology has focused on the gp350 glycoprotein, which is known to be essential to EBV entry [197], because of its potent immunogenicity [198,199]. Zhang et al. designed a series of chimeric virus-like particle vaccines displaying three gp350 receptor binding domain peptides named 149-3A through 149-3E. Both 149-3A and 149-3B generated higher antibody titers than the control, soluble gp350ECD123. Antibodies generated neutralized EBV infection. The other three constructs did not possess the same level of immunogenicity [200].

A second effort explored the efficacy of combining gp350 with the four glycoproteins gB, gp42, gH, and gL in a pentavalent EBV-like particle (EBV-LP). These particles stimulated antibodies to all glycoproteins included in the pentamer, although increases in gH/gL and gp42 antibodies were not significant. UV-inactivated EBV did stimulate greater IgG production than the pentamer; however, the EBV-LP vaccine stimulated greater production of neutralizing antibodies than gp350 alone. These antibodies inhibited infection of both B cell and epithelial cell lines. [201].

Nanoparticle vaccines have recently garnered attention as a new vaccination modality capable of avoiding the risks posed by killed, inactivated, or live-attenuated vaccines without sacrificing vaccine efficacy [202]. Kang et al. examined a gp350-based nanoparticle vaccine for EBV using the protein scaffolds lumazine synthase (LS) and I3-01. The nanoparticles gp350D_123_-LS and gp350D_123_-I3-01 showed greater antigenicity than the gp350 monomer and the gp350Ectodomain-LS nanoparticle. In addition, the neutralizing antibody 72AI and the non-neutralizing antibody 2L10 bound gp350-based particles with greater affinity than the gp350 monomer. In addition, gp350D123-LS and gp350D123-I3-01 stimulated higher levels of neutralizing antibody than gp350 alone. The immune response to the nanoparticle vaccine was found to be Th2/humoral-dominant. Neutralizing antibodies were detected at 30 weeks post injection. In addition, injecting Cynomolgus macaques with gp350D_123_-LS resulted in higher antibody titers than gp350D_123_. Nanoparticles combined with the adjuvant MF59 stimulated a more robust humoral response than nanoparticles combined with the adjuvant aluminum hydroxide [203].

### 5.2. Vaccination in EBV-Related Cancers

Given the association between EBV and cancers, attempts have been made to establish vaccine-based oncologic therapies. For example, a recent clinical trial examined the efficacy of inoculating nasopharyngeal carcinoma patients with dendritic cells pulsed with LMP2. Patients tolerated the series of three vaccines well. The study showed that 18 of the 29 patients demonstrated an augmented cytotoxic T cell response to LMP2 relative to the cytotoxic T cell response prior to vaccination. While improved responses were seen in all stages of nasopharyngeal carcinoma, response improvement was strongest in earlier stages. Patients who did not respond had decreased expression of CD83, CD86, and DC-DR on dendritic cells. Of the seven patients who died during the five-year follow-up period, all but one were non-responders to the vaccine. One of the seven enrolled in the trial was diagnosed with stage II disease, four with stage III disease, and two with stage IV disease. This data indicates that cytotoxic T cell responses to LMP2 may play a role in controlling nasopharyngeal carcinoma [204].

Prime–boost immunization refers to the practice of immunizing recipients with an immunogen, then either giving the same or a different immunogen as a booster [205]. This technique has recently been examined for efficacy in the setting of EBV-related cancers. Vaccination regimens examined included αDEC-E1 (recombinant antibodies that use DEC205 to target EBNA-1 to dendritic cells) plus Adeno–E1-LMP (a CD8+ T cell primer), Adeno–E1-LMP plus MVA-IiE1 (EBNA-1 without Gly/Ala repeats but with an invariant chain domain), αDEC-E1, and Adeno–E1-LMP. Adeno–E1-LMP plus αDEC-E1 was also given to assess the importance of the order in which the primer and booster were given. huDEC205-Tg mice were injected with EL4-E1 lymphoma cells. Prophylactic vaccinations were given two weeks before the lymphoma cell injection, while therapeutic vaccinations were given within seven days of ELF4-E1 cell injection. Eleven of thirteen mice were able to reject the tumor following prophylactic αDEC-E1 plus Adeno–E1-LMP or Adeno–E1-LMP plus MVA-IiE1 vaccination. These two vaccines were also the most effective when given therapeutically. αDEC-E1, Adeno–E1-LMP, and the inverse vaccination Adeno–E1-LMP plus αDEC-E1 slowed and inhibited tumor growth when given prophylactically; however, their efficacy differed significantly when given therapeutically. The inverse vaccine was equally as effective as αDEC-E1 plus Adeno–E1-LMP when given as a treatment, while αDEC-E1 and Adeno–E1-LMP showed only minimal effectiveness. Adeno–E1-LMP plus MVA-IiE1 was the only vaccine capable of decreasing nodal disease in both prophylactic and therapeutic settings.

The authors next examined the efficacy of αDEC-E1 plus Adeno–E1-LMP or Adeno–E1-LMP plus MVA-IiE1 in mice challenged with tumor cells designed to mimic Burkitt lymphoma cells expressing c-Myc. Adeno–E1-LMP plus MVA-IiE1 decreased levels of EBNA-1 DNA relative to mice injected with PBS, while EBNA-1 levels in mice injected with αDEC-E1 plus Adeno–E1-LMP were similar to the controls. Additionally, greater than 50% of mice vaccinated with Adeno–E1-LMP plus MVA-IiE1 had no evidence of disease, while 35% of mice vaccinated with αDEC-E1 plus Adeno–E1-LMP had no evidence of disease [206].

The association between latent EBV genes and cancer is well established [207,208,209]. Thus, it is unsurprising that latent EBV genes have been targets for vaccination efforts. Wojtak et al. recently designed synthetic consensus DNA vaccines targeting EBNA-1, LMP1, and LMP2A, as well as a vaccine combining the three. IFNγ produced in responses to latent protein peptide pools demonstrated that CD8+ T cells were the primary cells generating an immune response. Responses were more significant to EBNA-1 and LMP2A than LMP1 when injected in BALB/C, C57BL/6, and CD-1 mice. TC-1 cells were then made to express LMP2A and injected into C57BL/6 mice. Treatment with the LMP2A vaccine reduced tumor volume and increased the rate of tumor shrinkage when compared to the control [210].

## 6. Concluding Remarks

EBV is a highly prevalent virus that is typically found in a latent state in infected cells. This review explores how different types of stressors facilitate EBV reactivation. In addition, mechanisms of latency, factors involved in reactivation, antiviral therapy, and recent advances in vaccinations for both general prophylaxis and as an oncologic therapy have been addressed.

Reactivation is becoming increasingly important in a clinical context. A solid understanding of the mechanisms and triggers underlying reactivation will be useful in preventing illnesses associated with EBV reactivation, including post-transplant lymphoproliferative disorder [211], liver damage [212], and oral hairy leukoplakia [14]. An equally solid understanding of how to stimulate reactivation is also of utility. For example, inducing EBV reactivation is being explored as a cancer therapeutic in EBV-associated tumors [213,214,215,216,217]. A thorough understanding of EBV reactivation will enable us to harness the potential therapeutic benefits of antiviral therapies [21], improve transplant care [150], and develop cancer prophylaxis and treatment [206,216].

## Figures and Tables

**Table 1:** Mechanisms Involved in EBV Latency.

Mechanism	Effect	References
ROS Expression	B cell immortalization	[104,105,106]
-	Required for normal LMP1 expression	[106]
-	STAT3 phosphorylation	[106,107]
BHLF1	Maintenance of type III latency	[108]
PAX5	EBNA-1 localization to oriP and TR-DNA	[119]
-	Association of transcription enhancers from oriP and TR-DNA	[119]
CAF1	Inhibits lytic gene expression and increases histone presence at multiple points on the EBV genome.	[120]
HIRA	Histone loader involved in maintaining latency	[120]
ATRX	Histone loader involved in maintaining latency	[120]
DAXX	Histone loader involved in maintaining latency	[120]
MYC	Acts on BZLF1 promoter to prevent oriLyt and TR-DNA from looping	[110]
SMC1A	Contributes to latency by promoting MYC expression	[110]
Facilitated Chromatic Transcription Complex	Contributes to latency by promoting MYC expression	[110]
CXCR4	Maintenance of latency; stimulates LMP2A and EBNA-1	[112]
SUMOylation/SIM-interacting motifs	Facilitates oriP mini genome maintenance and the binding of EBNA-1 to His-tagged SUMO1 and SUMO2 proteins	[116]
-	EBNA-1 targets proteins with SUMO2 modifications for degradation	[116]
-	Inhibits BZLF1 expression	[116]
miRNAs	Inhibition of B cell receptor activation by diminishing **NF-κB** and/or AP-1 signaling	[123]

**Table 2:** Stimulators of EBV Reactivation.

Factor	Mechanism	Reference
KLF4, BLIMP1	Stimulates LMP1, then works with LMP1 to activate BZLF1 and BRLF1 expression	[127,128,129]
BCR signaling	Stimulates EBV reactivation	[130]
PI3K signaling	Stimulates BZLF1 promoter	[139]
IRF8	Influences caspase activity and KAP1 cleavage	[141]
Chaetocin	Stimulates ROS production	[143]
EBV upregulation of NADPH oxidase genes	Stimulates ROS production	[146]
NRF2 upregulation	Prevents ROS-mediated cell death	[146]
LMP1	Stimulates ROS production	[146]
LMP1 and LMP2A	Promote NRF2 production and translocation to nucleus	[147]
AKT	Involved in LMP1/LMP2A/NRF2 signaling	[148]
miRNA-18a	Stimulates transition from G1 to S phase	[148]
Hypoxia	Stimulates reactivation	[148]
Mycophenolate mofetil (immunosuppressant)	Impairs Vδ2+ T cell recovery	[155]
Syphilis	Cross-links TLR2 and BCR	[157]
HPV	E6 and E7 ** stimulates reactivation	[158,159]
As_2_O_3_ *	Stimulates BZLF1 transcription	[166,167]
Radiation	Induces BZLF1 and BLLF	[168]

* Conflicting evidence. ** E7 inhibited reactivation in immortalized keratinocytes.

## Abbreviations

BTdCPU	1-(benzo[d][1–3]thiadiazol-6-yl)-3-(3,4-dichlorophenyl)urea
TPA	12-0-tetradacanoyl-phorbol- 13-acetate
ATF4	Activating transcription factor 4
AKT	AKT serine/threonine kinase
ATRX	Alpha thalassemia/mental retardation syndrome X-linked chromatin remodeler
ATM	ATM serine/threonine kinase
BLNK	B cell linker protein
BCR	B cell receptor
BART	BamHI fragment A rightward transcript
BLIMP1	B-lymphocyte-induced maturation protein 1
BLK	B-lymphoid tyrosine kinase
Btk	Bruton’s tyrosine kinase
cP	C promoter
CTCF	CCCTC-binding factor
sgCHAF1B	CHAF1B single guide RNA
CAF	Chromatin assembly factor
CHAF1A	Chromatin assembly factor 1 subunit A
CHAF1B	Chromatin assembly factor 1 subunit B
3C	Chromatin conformation capture
ChIP	Chromatin immunoprecipitation
CD21	Complement receptor type 2
COVID-19	Coronavirus disease-19
CBF	C-promoter binding factor
CXCR4	C-X-C motif chemokine receptor 4
CMV	Cytomegalovirus
DAXX	Death domain-associated protein
dATP	Deoxyadenosine triphosphate
DNMT3A	DNA methyltransferase 3 alpha
PKR	Double-stranded RNA-dependent protein kinase
EBV-LP	EBV-like particle
EGFRK	Epidermal growth factor receptor-associated tyrosine kinase
EBNA	Epstein-Barr nuclear antigen
EBV	Epstein-Barr Virus
EBER	Epstein-Barr virus-encoded small RNA
eIF2α	Eukaryotic translation initiation factor 2 alpha
ERK	Extracellular signal-related kinase
FACT	Facilitated chromatin transcription
FPAX5	Flag epitope-tagged PAX5
GCN2	General control non-derepressible 2
GSK-3β	Glycogen synthase kinase 3β
g	Glycoprotein
Gy	Gray
GRB2	Growth factor receptor-bound protein 2
IC_50_	Half-maximal inhibitory concentration
HO-1	Heme oxygenase-1
HRI	Heme-regulated eIF2α kinase
HBV	Hepatitis B virus
H3K27me3	Histone 3 lysine 27 trimethyl
H3K4me3	Histone 3 lysine 4 trimethyl
H3K9me3	Histone 3 lysine 9 trimethyl
HHV	Human herpesvirus
HPV	Human papillomavirus
H_2_O_2_	Hydrogen peroxide
HPA	Hypothalamic-pituitary-adrenal
Ig	Immunoglobulin
ITAM	Immunoreceptor tyrosine-based activation motifs
IGF1	Insulin like growth factor 1
ISR	Integrated stress response
ICU	Intensive care unit
IRF	Interferon regulatory factor
IL	Interleukin
KSHV	Kaposi sarcoma-associated herpesvirus
KLF4	Krüppel-like factor 4
LMP	Latent membrane protein
oriP	Latent replication origin
lncRNA	long noncoding RNA
LS	Lumazine synthase
PCAF	Lysine acetyltransferase 2B
miRNA	microRNA
MAP	Mitogen-activated protein
ME/CFS	Myalgic encephalomyelitis/chronic fatigue syndrome
NACA	N-acetylcysteine amide
NQO-1	NAD(P)H-quinone oxidoreductase 1
NOX	NADPH oxidase
NPC	Nasopharyngeal carcinoma
Nrf2	Nuclear factor erythroid 2-related-factor 2
NF-κB	Nuclear factor kappa B
NFAT	Nuclear factor of activated T cells
NCL	Nucleolin
ORF	Open reading frame
oriLyt	Origin of lytic replication
GSSG	Oxidized glutathione
PAX	Paired box
PBMC	Peripheral blood mononuclear cell
PI3K	phosphatidylinositol 3-kinase
PLCγ2	Phospholipase Cγ2
PERK	PKR-like ER kinase
PDGFRK	Platelet-derived growth factor receptor-associated tyrosine kinase
PARP	Poly(ADP-ribose) polymerase
PTLD	Posttransplant lymphoproliferative disorder
PKC	Protein kinase C
Qp	Q promoter
RAC1	Ras-related C3 botulinum toxin substrate 1
RBBP4	RB binding protein 4
ROS	Reactive oxygen species
RT-PCR	Reverse transcription polymerase chain reaction
SRF	Serum response factor
shRNA	Short hairpin RNA
STAT3	Signal transducer and activator of transcription 3
siRNA	Small interfering RNA
SUMO	Small ubiquitin-related modifier
SOS1	SOS Ras/rac guanine nucleotide Exchange factor
SYC	Spleen tyrosine kinase
SUPT16H	SPT16 homolog, facilitates chromatin remodeling subunit
STUB1	STIP1 homology and U-box containing protein 1
SMC1A	Structural maintenance of chromosomes 1A
SSRP1	Structure specific recognition protein 1
SIMs	SUMO interaction motifs
Th2	T helper 2 cells
TAF	Tenofovir alafenamide
TDF	Tenofovir disoproxil fumarate
TET2	Tet methylcytosine dioxygenase 2
TLR	Toll-like receptor
GSH	Total glutathione
TRIB3	Tribbles pseudokinase 3
KAP1	Tripartite motif containing 28
UEV	Ubiquitin E2 variant
USP7	Ubiquitin specific peptidase 7
UV	Ultraviolet
VCA	Viral capsid antigen
